# GCT-BCLN: a bidirectional closed-loop network for nondestructive detection of rice seed vigor using hyperspectral imaging

**DOI:** 10.3389/fpls.2026.1923668

**Published:** 2026-08-17

**Authors:** Linyu Yan, Ending Xu, Xiangan Meng, Shuxin Jiang, Juan Liao, Yi Ren, Xinchun Zhan, Yu Zou

**Affiliations:** 1 College of Electronics and Electrical Engineering, Anhui Agricultural University, Hefei, China; 2 Anhui Province Key Laboratory of Rice Germplasm Innovation and Molecular Improvement, Rice Research Institute, Anhui Academy of Agricultural Sciences, Hefei, China; 3 College of Agriculture, Anhui University of Science and Technology, Fengyang, China

**Keywords:** CNN, gated recurrent units, hyperspectral imaging, rice seed vigor detection, transformer

## Abstract

Rice seed vigor is a key determinant of germination performance and final crop yield, making its rapid and non-destructive assessment essential for seed quality evaluation. Conventional vigor detection methods are often destructive, labor-intensive, and time-consuming. Hyperspectral imaging provides a promising non-destructive alternative, but hyperspectral data are typically high-dimensional, redundant, and susceptible to noise and scattering interference. Moreover, existing models still have limited ability to discriminate subtle spectral differences among seed vigor levels. To address these challenges, this study proposes a gated recurrent unit (GRU)-guided closed-loop CNN-Transformer network (GCT-BCLN) for accurate, non-destructive identification of rice seed vigor. The model establishes bidirectional information flow between CNN and Transformer via the GRU, enabling dynamic and synergistic optimization of local spectral features and global spectral representations. In addition, a combined preprocessing strategy integrating adaptive iteratively reweighted penalized least squares (AirPLS), Savitzky–Golay (SG) smoothing, and multiplicative scatter correction (MSC) was adopted to improve spectral quality. Experimental results showed that GCT-BCLN achieved a test accuracy of 0.9795 for hybrid indica rice, outperforming the CNN-Transformer fusion model by 1.37%. The model also achieved accuracies of 0.9793 and 0.9758 on conventional japonica rice and glutinous japonica rice, respectively, showing consistent performance across the three evaluated variety-specific datasets under the controlled experimental protocol. These results support the feasibility of GCT-BCLN for laboratory-scale, non-destructive discrimination of aging-induced rice seed categories under controlled conditions, while practical application requires further external validation.

## 1 Introduction

Rice is one of the world’s most important staple crops, and its yield and grain quality are closely related to global food security (Jin et al., 2022; Qi et al., 2023). In crop production, seed quality is a key determinant of field performance, among which seed vigor is a crucial physiological indicator affecting germination, seedling establishment, and ultimately crop productivity (Thakur et al., 2022). High-vigor seeds, characterized by stronger stress tolerance and growth potential, contribute to stable and high yields (Zhang et al., 2020). Therefore, accurate and efficient assessment of rice seed vigor is of great significance for seed breeding, processing, and storage management. Conventional methods, including standard germination tests, electrical conductivity measurements, and tetrazolium staining, can differentiate seed vigor to some extent, but they are generally limited by laborious procedures, long detection cycles, and destructive sampling requirements (Xing et al., 2023). With the development of intelligent and precision agriculture, there is an increasing demand for non-destructive and accurate methods for rice seed vigor assessment.

Recent advances in hyperspectral imaging (HSI) have provided a promising non-destructive technique for seed vigor evaluation (Taheri et al., 2024). By combining spatial information, such as seed morphology and texture, with spectral signatures associated with molecular vibrations of internal biochemical constituents, HSI enables seed quality assessment without physical damage (Chen et al., 2024). Its potential has been demonstrated in several seed vigor studies. For example, Wu et al. (2022) applied HSI to detect rice seed vigor under sample-imbalanced conditions. Zhang et al. (2025) used HSI to predict the vigor of naturally aged Xishuangbanna cucumber seeds, and Shi et al. (2025) achieved non-destructive detection of maize seed vigor using a data-fusion-driven HSI approach. Nevertheless, hyperspectral data are typically characterized by high dimensionality (Liao and Wang, 2025), substantial redundancy (Yu et al., 2025), susceptibility to noise (Wang et al., 2025), and sample-specific variability, all of which hinder robust feature extraction and modeling. Conventional pipelines combining spectral preprocessing methods, such as first derivative (FD) (Hu et al., 2025), Savitzky-Golay (SG) smoothing (Liu et al., 2025), and standard normal variate (SNV) transformation (Ruttanadech et al., 2025), with traditional machine learning models, including partial least squares regression (PLSR) (Saavedra et al., 2024), support vector machine (SVM) (Yang et al., 2021), and Random Forest (RF) (Zhu et al., 2025), can improve seed vigor prediction to some extent. However, these methods usually depend on manual feature engineering and often fail to capture the complex nonlinear relationships between spectral information and seed vigor, resulting in limited generalization and reduced sensitivity to subtle vigor differences.

Deep learning, with its end-to-end feature learning capability, has shown strong potential for modeling high-dimensional and highly correlated hyperspectral data. In seed vigor detection, Li et al. (2025) proposed a multi-scale spectral attention residual network (MSARN) for common bean seed vigor assessment, achieving accuracy, precision, recall, and F1-score values of 98.75%, 98.97%, 98.80%, and 98.81%, respectively. Pang et al. (2021) combined CNNs with a long short-term memory (LSTM) network to model the vigor of *Styphnolobium japonicum* seeds, achieving recognition accuracy above 90%. Despite these advances, conventional CNNs are limited by their kernel-size-dependent receptive fields and tend to emphasize local spectral patterns while underrepresenting long-range spectral relationships. This limitation restricts their ability to model complex spectral signatures associated with seed aging and biochemical heterogeneity. To jointly capture local and global information, recent studies have introduced Transformer architectures, which model long-range dependencies through self-attention. Zheng et al. (2025) developed a CNN-Transformer fusion model for rice variety classification using reconstructed hyperspectral images, achieving 99.413% accuracy and markedly outperforming a standalone CNN. Liu et al. (2025) proposed a dual-feature transformation fusion transformer (DFTFT), which explicitly models global spectral associations for wheat and maize leaf disease and quality grade classification, improving classification accuracy by 5.2%–7.8% compared with single-CNN baselines. Chakrabarty et al. (2024) designed an interpretable lightweight CNN-Transformer hybrid model for rapid rice leaf disease identification, achieving an accuracy of up to 97%.

Although CNN-Transformer hybrid models have shown advantages over conventional CNNs in crop hyperspectral recognition and classification, rice seed vigor detection remains challenging. First, spectral differences among seeds at different aging stages are often subtle, requiring models with strong sensitivity to fine inter-class variations. Second, the curved surface morphology of rice seeds induces scattering heterogeneity, causing spectra from seeds with the same vigor level to be affected by sample-specific physical differences. Third, varietal diversity leads to differences in spectral baselines and characteristic peaks, further complicating feature learning. Most existing CNN-Transformer fusion models adopt cascaded serial architectures (Liu et al., 2023), in which features are transmitted unidirectionally and statically. In such designs, local feature extraction is weakly guided by global spectral representations, while global modeling provides limited feedback for refining local features. This restricts model robustness under subtle vigor differences and spectral interference. Therefore, dynamic and synergistic optimization of local and global features is needed to improve the adaptability of hyperspectral models for rice seed vigor assessment. Bidirectional feedback has been shown to enhance feature interaction in other fields. Wang et al. (2022) proposed a lightweight bidirectional feedback network for image super-resolution, reducing parameters while improving reconstruction quality. Jiang et al. (2023) incorporated encoder–decoder bidirectional transmission with dense connections into U-Net (BiDFDC-Net), improving skin lesion segmentation. In remote sensing, Ge et al. (2025) proposed a dual-stream spectral interactive network with multi-scale enhancement, cross-attention complementarity, and cross-layer interaction for salient object detection. However, such bidirectional feedback mechanisms remain rarely explored in agricultural hyperspectral analysis, particularly for seed vigor assessment. Collectively, these studies indicate that reciprocal feature transmission can improve representation refinement, but existing approaches have not yet provided a stateful feedback mechanism for iterative interaction between local and global representations of wavelength-ordered hyperspectral signals.

To address this limitation, this study proposes a GRU-guided bidirectional closed-loop CNN-Transformer network (GCT-BCLN) for rice seed vigor identification. In this framework, the term bidirectional describes the two-way information flow between the CNN and Transformer: CNN-derived local spectral features are propagated forward for global dependency modeling, whereas Transformer-derived global representations are fed back to refine CNN-based local feature extraction. By contrast, the term closed-loop refers to the recurrent use of this two-way pathway, whereby feedback-refined local features re-enter the GRU-Transformer pathway in subsequent iterations until the prediction distribution stabilizes. The GRU is adopted as the feedback carrier because hyperspectral bands form an ordered sequence, and its gated hidden state can retain wavelength-dependent contextual information while selectively integrating the current global representation with information from previous feedback iterations. Through this iterative local-global interaction, GCT-BCLN is designed to enhance the representation of subtle vigor-related spectral differences and improve robustness to spectral interference. The proposed framework provides a useful methodological basis for non-destructive rice seed vigor assessment using hyperspectral data.

## 2 Materials and methods

**Figure 1:**
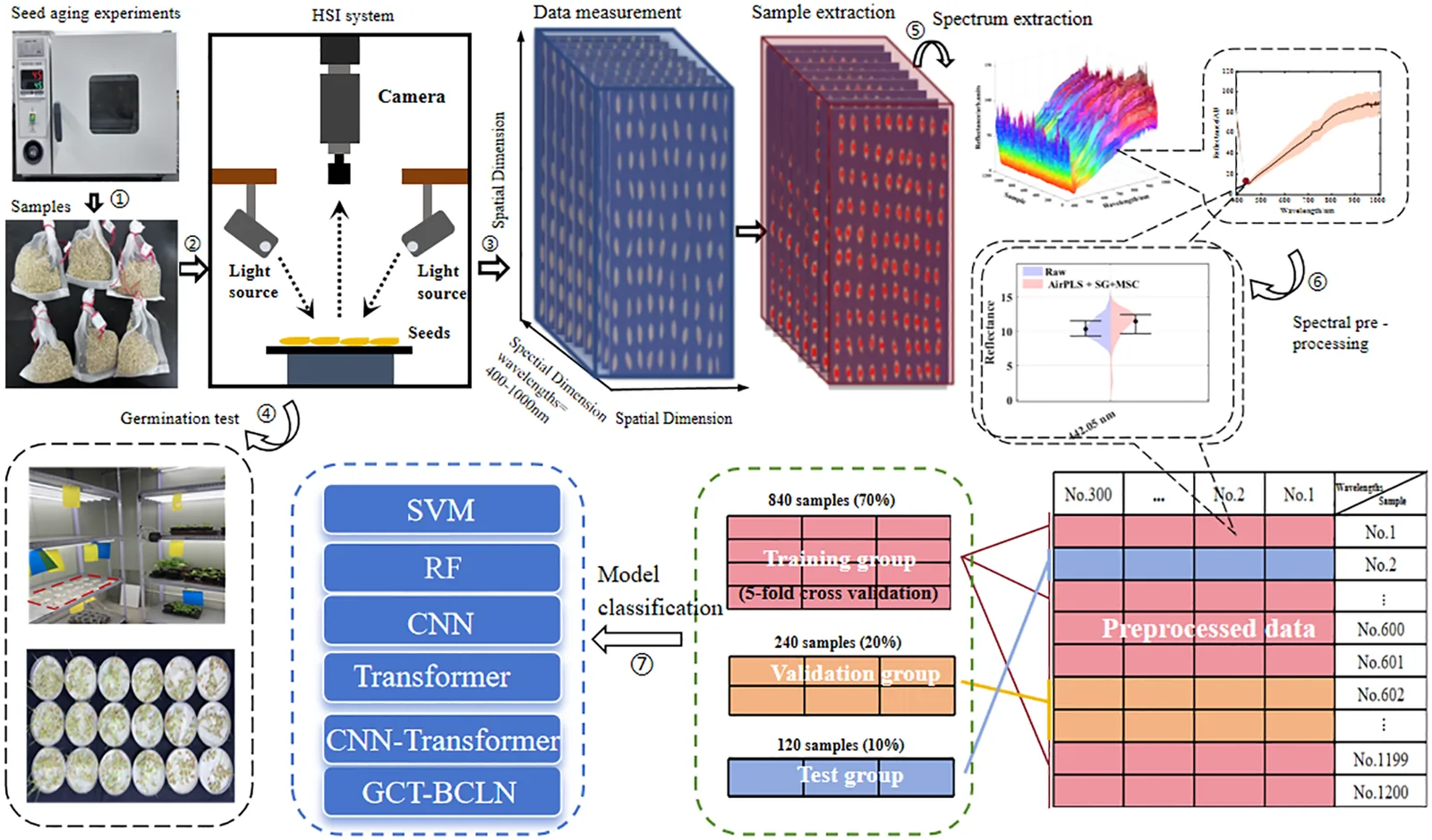
Overall experimental and modeling workflow.

To systematically achieve non-destructive rice seed vigor detection, this study established an integrated workflow encompassing sample preparation, hyperspectral data acquisition and processing, spectral preprocessing, and model construction, as illustrated in **Figure 1**. Sample preparation generated seed samples with different vigor levels for subsequent data acquisition. Hyperspectral imaging and spectral preprocessing provided high-quality spectral inputs for model development, while standard germination tests were conducted to establish reference labels for vigor assessment. Finally, the proposed model was constructed to map spectral features to corresponding vigor levels. The operational procedures and principles of each stage are described in detail in the following sections.

### 2.1 Preparation of rice seed samples with different vigor levels

This study employed an artificial accelerated aging method (Li et al., 2024) to prepare rice seed samples with aging-induced vigor categories under controlled laboratory conditions. Three rice varieties were used in this study, including hybrid indica rice, conventional japonica rice, and glutinous japonica rice. All seeds were harvested in 2024 and provided by the Rice Research Institute of the Anhui Academy of Agricultural Sciences, China. For each variety, diseased, damaged, and empty grains were removed, and 1,200 plump, healthy seeds with uniform morphology were selected. Therefore, a total of 3,600 individual seeds were included in the experiments. The hybrid indica rice dataset was used as the primary dataset for model development and comparative evaluation, whereas the conventional japonica rice and glutinous japonica rice datasets were used as additional variety-specific evaluation datasets. Before aging, the seeds were surface-sterilized by immersion in 1% sodium hypochlorite (NaClO) solution for 15 min, thoroughly rinsed with sterile deionized water at least five times, and air-dried at room temperature until no visible surface moisture remained. Accelerated aging was conducted under controlled high-temperature and high-humidity conditions (Fantazzini et al., 2018). Briefly, 500 mL of sterile water was added to a glass desiccator, which was then placed in a dark incubator maintained at 45 °C. After the internal environment stabilized at 45 °C and approximately 100% relative humidity, the seeds were placed in breathable nylon mesh bags, arranged flat on a perforated porcelain plate inside the desiccator, and exposed to the aging treatment. For each rice variety, the 1,200 seeds were equally divided into six aging-duration groups, with 200 seeds in each group. Samples were collected after 0 d (control), 1 d, 2 d, 3 d, 4 d, and 5 d of aging to generate seed lots with a controlled vigor deterioration gradient. Temperature and relative humidity inside the desiccator were continuously monitored to ensure treatment consistency. After aging, seeds from each treatment were transferred to room-temperature conditions for moisture equilibration, thereby minimizing the influence of moisture differences on subsequent hyperspectral measurements and germination testing. It should be emphasized that the accelerated-aging protocol was used to generate a controlled and reproducible vigor gradient for methodological development rather than to reproduce the complete deterioration process of naturally aged commercial seed lots. The six classification targets in this study were defined according to aging duration rather than commercially defined vigor grades. Therefore, the current dataset should be interpreted as a laboratory-scale dataset for methodological evaluation rather than as a direct representation of naturally aged commercial seed lots.

### 2.2 Hyperspectral data acquisition

**Figure 2:**
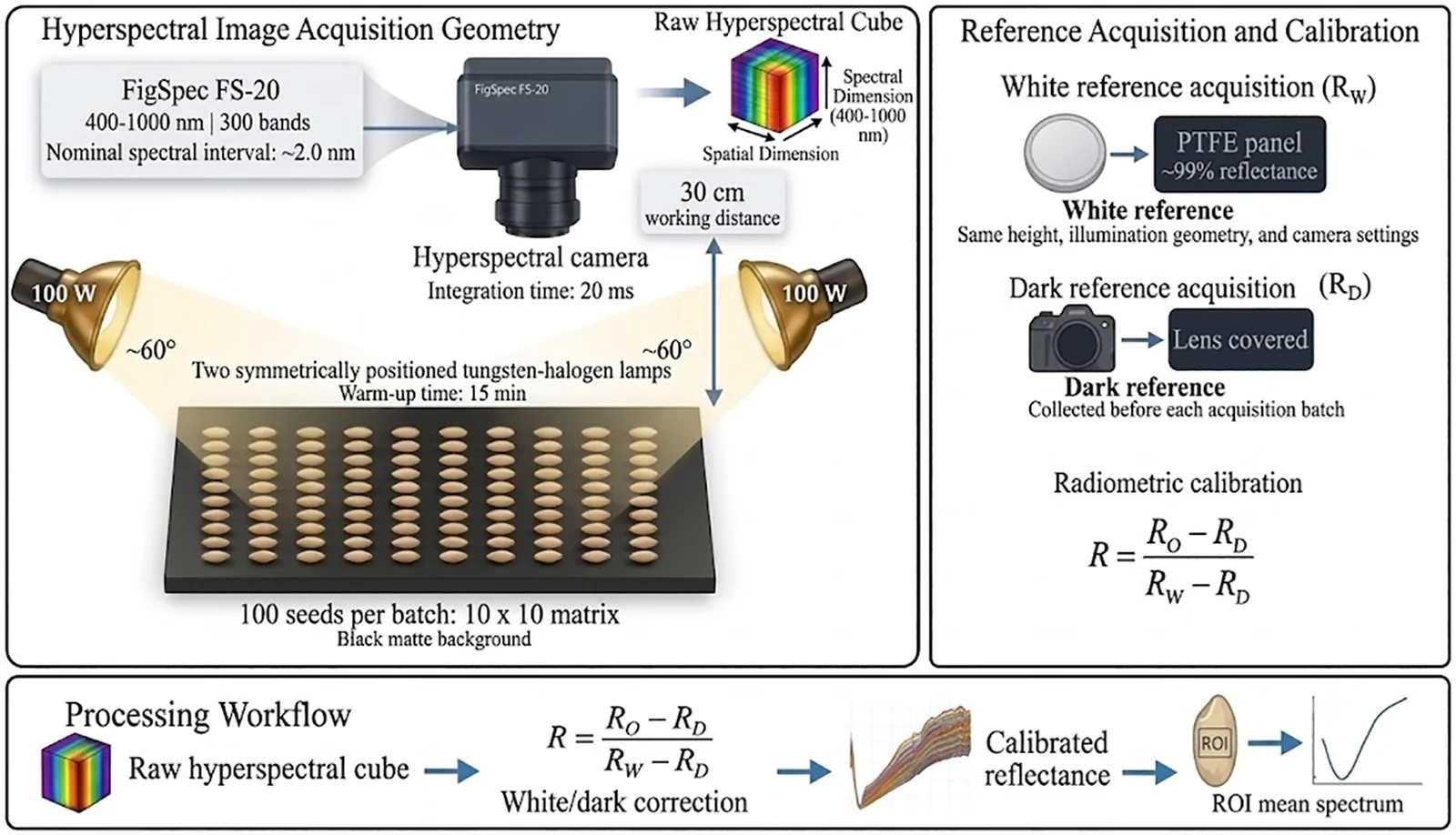
Hyperspectral image acquisition, radiometric calibration, and mean-spectrum extraction workflow.

Hyperspectral images of aged rice seeds were acquired using a FigSpec FS-20 hyperspectral imaging system, as illustrated in **Figure 2**. The system acquired 300 spectral bands over the wavelength range of 400–1000 nm, corresponding to a nominal spectral sampling interval of approximately 2.0 nm. The spatial dimensions of each hyperspectral image were 1920 × 1920 pixels. For each aging treatment, 200 seeds were imaged in two batches. In each batch, 100 seeds were arranged without overlap in a 10 × 10 matrix on a black matte background to minimize background reflection and facilitate subsequent ROI extraction. Illumination was provided by two 100 W tungsten-halogen lamps positioned symmetrically on both sides of the sample platform at an angle of approximately 60°. The hyperspectral camera was mounted vertically above the samples, with a lens-to-sample working distance of 30 cm. The integration time was set to 20 ms. Before image acquisition, the lamps were warmed up for 15 min to ensure stable illumination, and all acquisition parameters were kept constant throughout the experiment. Before each acquisition batch, white- and dark-reference images were collected for radiometric calibration. The white-reference image was acquired using a polytetrafluoroethylene (PTFE) diffuse-reflectance panel with a nominal reflectance of approximately 99%. The reference panel was placed under the same imaging geometry and camera settings as the seed samples. The dark-reference image was acquired with the camera lens completely covered. To reduce the effects of dark current, non-uniform illumination, and sensor noise, the raw hyperspectral images were radiometrically calibrated using the manufacturer-provided software (Chen et al., 2025). The corrected reflectance was calculated according to (Equation 1). After calibration, all subsequent data processing was performed using ENVI 5.6 software. For each seed, a fixed 10 × 10-pixel square region of interest (ROI) was manually positioned within the visually homogeneous central endosperm region, with its center located near the longitudinal axis of the seed. To reduce edge scattering and background interference, the ROI boundary was kept at least 3 pixels inside the visible seed contour. The embryo-bearing basal one-third of the seed, shadowed pixels, and visibly damaged areas were excluded. When positional adjustment was necessary, the ROI was translated within the central endosperm region without changing its dimensions. The mean reflectance spectrum of the 100 pixels within the ROI was used to represent the corresponding seed and was subsequently exported for spectral preprocessing and modeling. The same ROI-selection criteria were applied to all seeds and aging treatments.

(1) $$R=\frac{R_{\text{O}}-R_{\text{D}}}{R_{\text{W}}-R_{\text{D}}}$$

where R is the calibrated reflectance image, $R_{\text{O}}$ is the original hyperspectral image of the rice seeds, $R_{\text{D}}$ is the dark-reference image acquired with the camera lens completely covered, and $R_{\text{W}}$ is the white-reference image acquired from the PTFE reference panel under identical acquisition conditions.

### 2.3 Standard laboratory germination test

To quantify the physiological decline caused by accelerated aging, a standard laboratory germination test was conducted with reference to the principles of the International Rules for Seed Testing (International Seed Testing Association [ISTA], 2026). For each aging treatment, 150 seeds were tested in three replicates, with 50 seeds per replicate. Seeds were placed on moistened filter paper in 90-mm Petri dishes and incubated in a controlled-environment chamber at 30 ± 0.5 °C and 60 ± 5% relative humidity (RH) for 14 days. Sterile distilled water was added daily to maintain adequate moisture, and germination status was recorded daily. A seed was considered germinated when the radicle protruded through the seed coat and reached a length equal to or greater than half of the seed length (Zhang et al., 2020). After 14 days of incubation, the germination percentage was calculated for each replicate, and the mean value of the three replicates was used as the final germination percentage for the corresponding aging treatment. The six class labels used for spectral modeling corresponded to the aging durations (0–5 d), whereas germination percentage was used as a group-level physiological indicator to verify the progressive decline in germination performance. Therefore, the classification results should be interpreted as discrimination among aging-induced vigor categories under controlled laboratory conditions, rather than as direct measurements of the absolute vigor of naturally aged commercial seeds.

### 2.4 Dataset partitioning and spectral preprocessing

For each rice variety, 1,200 seed-level spectra were obtained, corresponding to six aging-duration categories, with 200 spectra in each category. The hybrid indica rice dataset was used as the primary dataset for model development and comparative evaluation, whereas the conventional japonica rice and glutinous japonica rice datasets were used as additional variety-specific evaluation datasets. To ensure balanced class distribution and reduce evaluation bias, the 200 spectra from each aging-duration category were randomly divided into training, validation, and test subsets at a ratio of 7:2:1, yielding 140, 40, and 20 spectra per level, respectively. Consequently, each variety-specific dataset contained 840 training spectra, 240 validation spectra, and 120 test spectra. The global random seed was fixed at 42 during dataset partitioning and model weight initialization to improve experimental reproducibility. Five-fold cross-validation was conducted only on the training set of the primary hybrid indica rice dataset to support hyperparameter tuning and assess training stability. The validation set was used for model selection, early stopping, and ablation analysis, whereas the test set was strictly reserved for final performance evaluation. For the conventional japonica rice and glutinous japonica rice datasets, model training, validation, and testing were performed separately within each variety-specific dataset using the same dataset-partitioning procedure, spectral preprocessing strategy, model architecture, and hyperparameter settings. Therefore, these two datasets were used to examine whether the same modeling workflow produced consistent within-dataset performance across different rice varieties, rather than to claim external cross-variety generalization.

To improve hyperspectral data quality, a combined preprocessing strategy integrating adaptive iteratively reweighted penalized least squares (AirPLS) (Cui et al., 2025), SG smoothing, and multiplicative scatter correction (MSC) (Guan et al., 2024) was adopted. These preprocessing steps were designed to reduce baseline drift, high-frequency noise, and multiplicative scattering effects, thereby providing more reliable spectral inputs for vigor modeling. To avoid information leakage, all preprocessing parameters requiring data-driven estimation, particularly the MSC reference spectrum, were determined using only the training set and then applied to the validation and test sets. Raw spectra are often affected by baseline offsets caused by instrumental variations and illumination instability. Therefore, AirPLS was first applied for baseline correction. Based on a penalized least squares framework with iterative reweighting, AirPLS separates complex baseline shifts from informative spectral features, thereby restoring absorption characteristics while preserving useful spectral information (Zhu et al., 2022). The spectral vector, fitted baseline, penalized least-squares objective function, iterative weight update, and corrected spectrum are defined in (Equations 2–6), respectively. The baseline-corrected spectra were then processed using SG smoothing, which applies local polynomial fitting within a moving window to suppress high-frequency noise while preserving the overall spectral profile and characteristic absorption peaks (Wang et al., 2025). Finally, MSC was applied to reduce scattering effects caused by differences in seed surface morphology and optical path variation. By regressing each spectrum against the mean reference spectrum, MSC corrects additive and multiplicative scattering effects and improves the comparability of spectra among samples, thereby enhancing the extraction of vigor-related information.

(2) $$y={\left[y_{1},y_{2},\cdot \cdot \cdot ,y_{n}\right]}^{T}$$

(3) $$b={\left[b_{1},b_{2},\cdot \cdot \cdot ,b_{n}\right]}^{T}$$

(4) $$F=min{\left[\sum_{i=1}^{n}w_{i}{\left(y_{i}-b_{i}\right)}^{2}+\lambda \sum_{i=1}^{n-2}(b_{i+2}-2b_{i+1}+b_{i}\right)}^{2}]$$

(5) $$w_{i}=\left\{ \begin{array}{l} 0,\;y_{i}\geq b_{i}^{t-1}\\ {\text{e}}^{\frac{t(y_{i}-b_{i}^{t-1})}{\left|d^{-}\right|}},\;y_{i}<b_{i}^{t-1} \end{array}\right.$$

(6) $$y_{corrected}=y-\hat{b}$$

where *y* is the original spectral vector, *b* is the fitted baseline, *F* is the penalized least squares function whose minimum is sought to determine *b*, λ is the penalty parameter, *w_i_* is the weight vector controlling the closeness of the fit between the baseline and the spectral vector at each sampling point, $d^{-}$ contains the negative elements of the difference *y*-*b*, *t* is the iteration number, *ycorrected*.is the corrected spectral vector.

### 2.5 Model development

#### 2.5.1 Overall network architecture

**Figure 3:**
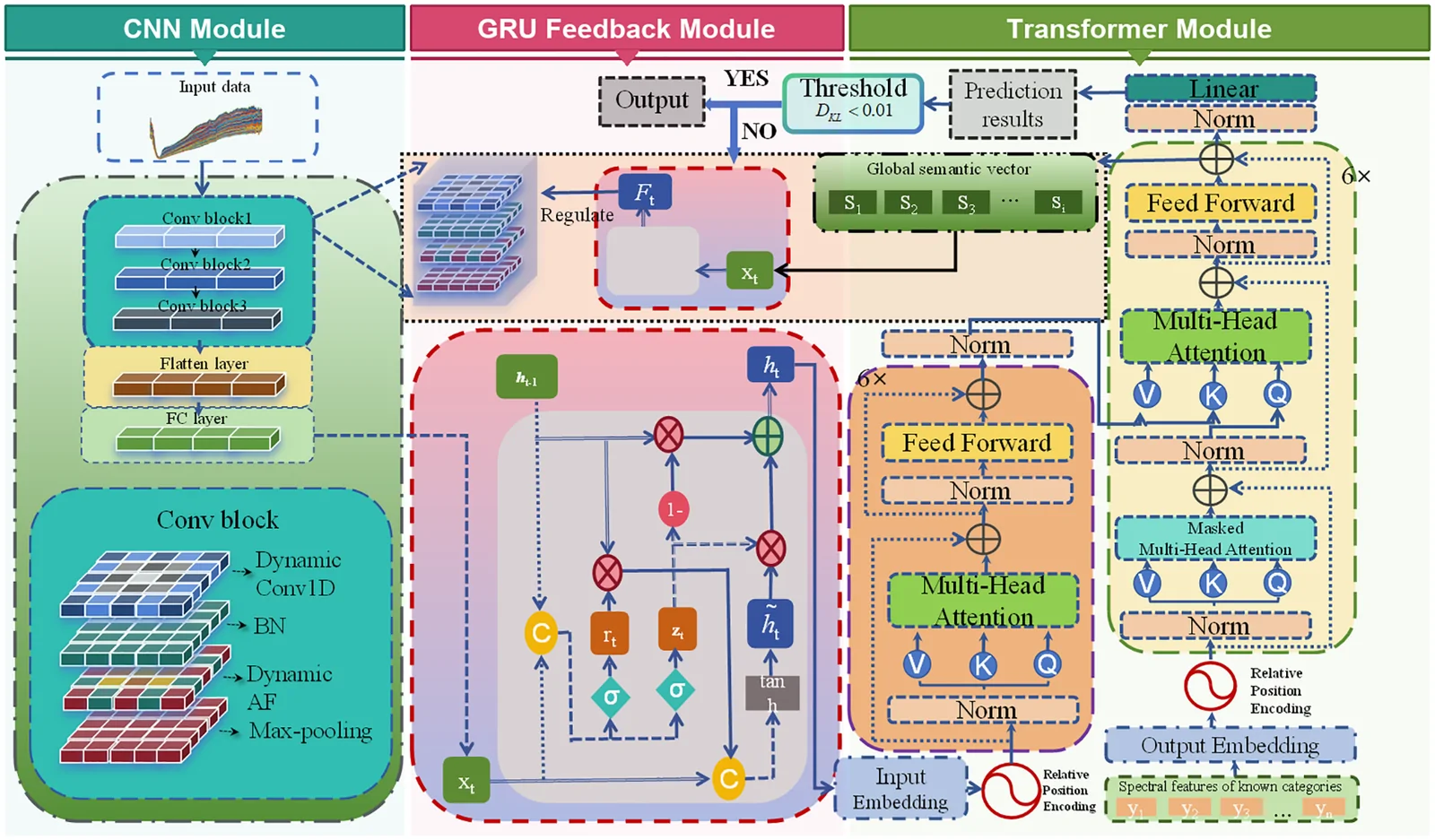
Overall architecture of the proposed GCT-BCLN.

This study proposes a GRU-guided closed-loop CNN-Transformer network (GCT-BCLN) for rice seed vigor detection. As shown in **Figure 3**, the proposed framework consists mainly of a CNN-based local feature extraction module, a GRU-based spectral sequence modeling and feedback module, and a Transformer-based global spectral modeling module. Specifically, the CNN is employed to extract fine-grained local features, which are then reordered by wavelength and fed into the GRU to capture short-range inter-band dependencies. The Transformer subsequently applies relative position encoding and multi-head self-attention to model long-range spectral dependencies, yielding global spectral features and prediction probabilities. Different from conventional cascaded structures, GCT-BCLN further constructs a bidirectional closed-loop feedback pathway. Through this closed-loop interaction among local extraction, spectral sequence encoding, global dependency modeling, and feedback regulation, the proposed GCT-BCLN achieves coordinated optimization of local and global features, thereby improving the discrimination of subtle vigor differences and enhancing robustness to scattering-induced spectral heterogeneity.

#### 2.5.2 CNN-based local feature extraction

The CNN local feature extraction module is designed to extract discriminative multi-scale local detail features in the rice seed hyperspectral data. As shown in **Figure 3**, this module consists of three cascaded convolutional blocks. Each block includes a dynamic Conv1D layer, batch normalization (BN), a dynamic hybrid activation function pool, and a max-pooling layer. Through hierarchical feature transformation, the module progressively captures fine-grained, band-localized spectral patterns that are closely associated with subtle differences in seed vigor. The initial one-dimensional convolutional kernel sizes of the three blocks are set to 5, 4, and 3, respectively, forming a coarse-to-fine receptive-field hierarchy along the spectral dimension. To further enhance the sensitivity of the CNN to vigor-related spectral variations, a dynamically adjustable convolutional kernel mechanism is introduced. In this mechanism, the convolutional parameters are updated not only according to the gradient of the total loss, but also under the guidance of the GRU-derived feedback signal. The dynamic update rule is defined in (Equations 7, 8).

(7) $$\theta_{t+1}^{k}=\theta_{t}^{k}+\partial \cdot z_{t}⨀\nabla_{\theta_{t}^{k}}L_{tatal}+\beta \cdot r_{t}⨀Map\left(F_{t}\right)⨀S\left(\lambda_{t}^{k}\right)$$

(8) $$\theta_{t}^{k}=\left\{W_{t}^{k},b_{t}^{k}\right\}$$

where $\theta_{t}^{k}$ represents the parameter set of the *k*-th convolutional kernel at the *t*-th iteration, including the weight matrix $W_{t}^{k}$ and the bias vector $b_{t}^{k}$. $W_{t}^{k}$ determines the spectral response pattern of the convolutional kernel, while $b_{t}^{k}$ provides channel-wise bias compensation. $\nabla_{\theta_{t}^{k}}L_{tatal}$ denotes the gradient of the loss function with respect to the parameters $\theta_{t}^{k}$ , ensuring the kernel updates towards minimizing error. $F_{t}$ is the feedback feature generated by the GRU-mediated feedback pathway, and Map(·) is a fully connected mapping function responsible for transforming the feedback signal into a parameter adjustment term compatible with the convolutional kernel. $S\left(\lambda_{t}^{k}\right)$ is the spectral sensitivity function, defined as: $S\left(\lambda_{t}^{k}\right)=\frac{1}{1+e^{-\gamma (\lambda_{t}^{k}-\lambda_{0})}}$ , where $\lambda_{t}^{k}$ represents the primary response wavelength of the k-th convolutional kernel in the *t*-th iteration, $\lambda_{0}$ is a baseline wavelength, and ∂, β, γ are hyperparameters dynamically adjusted based on training accuracy. Additionally, $z_{t}$ and $r_{t}$ represent the outputs of the update gate and reset gate in the GRU, respectively, controlling the information fusion ratio between the historical state and the current input.

Activation functions provide the nonlinear transformations required for CNNs to model complex spectral relationships (Sayın et al., 2025). To improve adaptability and robustness in hyperspectral feature learning, a dynamic hybrid activation function pool is further introduced. Instead of using a single fixed activation function, this mechanism adaptively fuses multiple candidate activation functions through learnable weights. The contribution of each activation function is dynamically adjusted according to the current input feature distribution and the GRU feedback signal, as formulated in (Equations 9, 10).

(9) $$w_{t+1}^{i}=\frac{w_{t}^{i}\times e^{\eta \times Corr\left(f_{i}\left(x_{t}\right),F_{t}\right)}\times g_{t}}{\sum w_{t}^{j}\times e^{\eta \times Corr\left(f_{j}\left(x_{t}\right),F_{t}\right)}\times g_{t}}$$

(10) $$f(x)=\sum w_{t}^{i}f_{i}(x)$$

where $w_{t}^{i}$ denotes the weight of the *i*-th activation function at the *t*-th iteration. $Corr\left(f_{i}\left(x_{t}\right),F_{t}\right)$ is the Pearson correlation coefficient, measuring the correlation between the activation function output $f_{i}\left(x_{t}\right)$ and the feedback signal $F_{t}$. A larger correlation value assigns a higher contribution to the corresponding activation function. $g_{t}$ is the feature complexity factor, where a larger value indicates stronger feature nonlinearity. $f_{i}(x)$ is the *i*-th activation function. η is a hyperparameter that regulates the influence strength of the correlation on the weight.

#### 2.5.3 GRU-based spectral sequence encoding

The gated recurrent unit (GRU) (Li et al., 2025) is an efficient sequence modeling unit in which the update and reset gates jointly control information retention and noise suppression (Mahadi et al., 2024), providing stable representation learning for ordered sequential data (Zhang et al., 2022). In this study, the local feature vectors extracted by the CNN are reorganized according to wavelength order from 400 to 1000 nm and treated as a spectral sequence. Specifically, the feature vector at the *t*-th step corresponds to the convolutional output for the *t*-th spectral band. The GRU then encodes ordered spectral sequence to capture short-range inter-band dependencies and generate a context-aware intermediate representation. These encoded features preserve local spectral correlations and spectral-order information, thereby providing informative priors for the subsequent Transformer module to model long-range dependencies across the full spectral range.

#### 2.5.4 Transformer-based global spectral modeling

Based on the GRU-encoded spectral sequence features, the Transformer module is used to capture long-range dependencies across spectral bands and generate global spectral representations. As shown in **Figure 3**, this module mainly consists of feature embedding, relative position encoding, multi-head self-attention, feed-forward layers, normalization layers, and multi-layer feature aggregation. Since hyperspectral bands are naturally arranged in ascending wavelength order, a sinusoidal relative position encoding strategy is introduced to replace conventional absolute positional encoding. This design explicitly characterizes the pairwise distance between spectral bands, thereby enhancing the model’s ability to perceive spectral continuity and inter-band structural relationships. The relative position encoding is formulated in (Equations 11, 12).

(11) $$R_{ij}\left(2k\right)=\sin(\frac{\left|i-j\right|}{10000^{\frac{2k}{d}}})$$

(12) $$R_{ij}\left(2k+1\right)=\cos(\frac{\left|i-j\right|}{10000^{\frac{2k}{d}}})$$

where $R_{ij}$ is the relative position encoding between the *i*-th and *j*-th bands, |i−j| represents their relative distance between the two bands. d is the encoding dimension (in this study, *d* = 300), *k* denotes the dimension index (satisfying $k\in \left[0,\frac{d}{2}-1\right]$).

For attention computation, a four-head multi-head self-attention mechanism is applied to the GRU-encoded spectral features. The query, key, and value matrices are obtained by linear projection of the input features. The relative position matrix (R), constructed from (Equations 11, 12), is incorporated into the attention score calculation to introduce pairwise spectral-distance information. The attention operation is defined in (Equation 13). In this study, six Transformer layers are stacked to perform hierarchical feature aggregation. Each layer contains multi-head self-attention, feed-forward transformation, residual connection, and normalization operations. Through this structure, the model integrates fine-grained local features extracted by the CNN, short-range inter-band dependencies encoded by the GRU, and long-range global associations modeled by the Transformer. The final global spectral representation is obtained through feature aggregation and then fed into a fully connected layer to predict the rice seed vigor level and its corresponding probability (P). This design provides a comprehensive spectral representation for accurate seed vigor classification.

(13) $$\text{Attention}(Q,K,V)=\text{Softmax}(\frac{QK^{T}+R}{\sqrt{d_{k}}})V$$

where *Q*, *K*, and *V* are the query, key, and value matrices, respectively. *d_k_* is the feature dimension of the key vector, and *R* is the relative position encoding matrix.

#### 2.5.5 GRU-based closed-loop feedback

To enable dynamic interaction between local feature extraction and global spectral modeling, this study introduces a GRU-based closed-loop feedback mechanism between the CNN and Transformer modules. In this mechanism, the global spectral representation generated by the Transformer is encoded into a feedback signal, which is then used to adaptively regulate the CNN feature extraction process. This design establishes an iterative feedback loop between local spectral feature learning and global dependency modeling. Specifically, at the t-th iteration, the global spectral vector S output by the Transformer is fed into a GRU together with the previous hidden state. The update gate *z_t_*, reset gate *r_t_*, candidate hidden state *h̃_t_*, and feedback signal *F_t_* are defined in Equations 14–17, respectively. The update gate controls the incorporation of current global spectral information, while the reset gate regulates the contribution of the previous hidden state. The resulting feedback signal *F_t_* is then used to guide the adaptive update of the CNN module. In particular, *F_t_* is mapped to the convolutional parameter adjustment term in (Equation 7), thereby modulating the dynamic convolutional kernels. Meanwhile, *F_t_* also participates in the correlation-based weighting strategy of the dynamic hybrid activation pool in (Equation 9), enabling the network to assign greater importance to activation responses that are more consistent with the current global spectral information. After feedback regulation, the refined local features are again passed through the GRU spectral encoder and the Transformer global modeling module to update the global representation and prediction probability distribution. This iterative procedure continues until the Kullback-Leibler (KL) divergence between two consecutive prediction distributions is lower than a predefined threshold. Once this convergence criterion is satisfied, the network outputs the final rice seed vigor classification result. The proposed closed-loop feedback mechanism differs from conventional cascaded structures, where information is usually transmitted in a one-way manner from local feature extraction to global modeling. In GCT-BCLN, CNN-extracted local features provide detailed spectral evidence for global spectral modeling, while Transformer-derived global representations feedback high-level contextual guidance to refine local feature extraction.

(14) $$z_{t}=\sigma (W_{z}\cdot \left[h_{t-1},S\right]+b_{z})$$

(15) $$r_{t}=\sigma (W_{r}\cdot \left[h_{t-1},S\right]+b_{r})$$

(16) $${\tilde{h}}_{t}=\tanh(W_{h}\cdot [r_{t}⨀h_{t-1},S]+b_{h})$$

(17) $$F_{t}=(1-z_{t})⨀h_{t-1}+z_{t}⨀{\tilde{h}}_{t}$$

where *S* is the global spectral vector output by the Transformer, $F_{t}$ is the feedback signal.

#### 2.5.6 Loss function and training configuration

To accommodate the closed-loop architecture and dynamic optimization mechanism of the GCT-BCLN model, a hybrid loss function is designed as shown in (Equation 18).

(18) $$L_{total}=\mu L_{CE}+(1-\mu )L_{KL}+\xi L_{reg}+\tau L_{dyn}$$

where *L_CE_* is the standard cross-entropy loss term, which aligns the predicted distribution with ground-truth labels and serves as the primary objective for the classification task. *L_KL_* is the KL divergence between the predicted distributions of two consecutive iterations, which effectively suppresses oscillations induced by feature variations in the closed-loop feedback by imposing a constraint on prediction consistency, thereby ensuring the stability of the iterative process. *L_reg_* is the L2 weight-decay regularization term applied to all trainable parameters to constrain parameter magnitude and reduce the risk of overfitting during training. *L_dyn_* is a specially designed regularize for the dynamic mechanism, which constrains the update magnitude of the dynamic convolutional kernels and the weight variations in the dynamic hybrid activation function pool. This prevents instability in feature extraction caused by excessive dynamic adjustments and ensures robust training of the network structure.

## 3 Results and discussion

### 3.1 Spectral analysis and preprocessing

**Figure 4:**
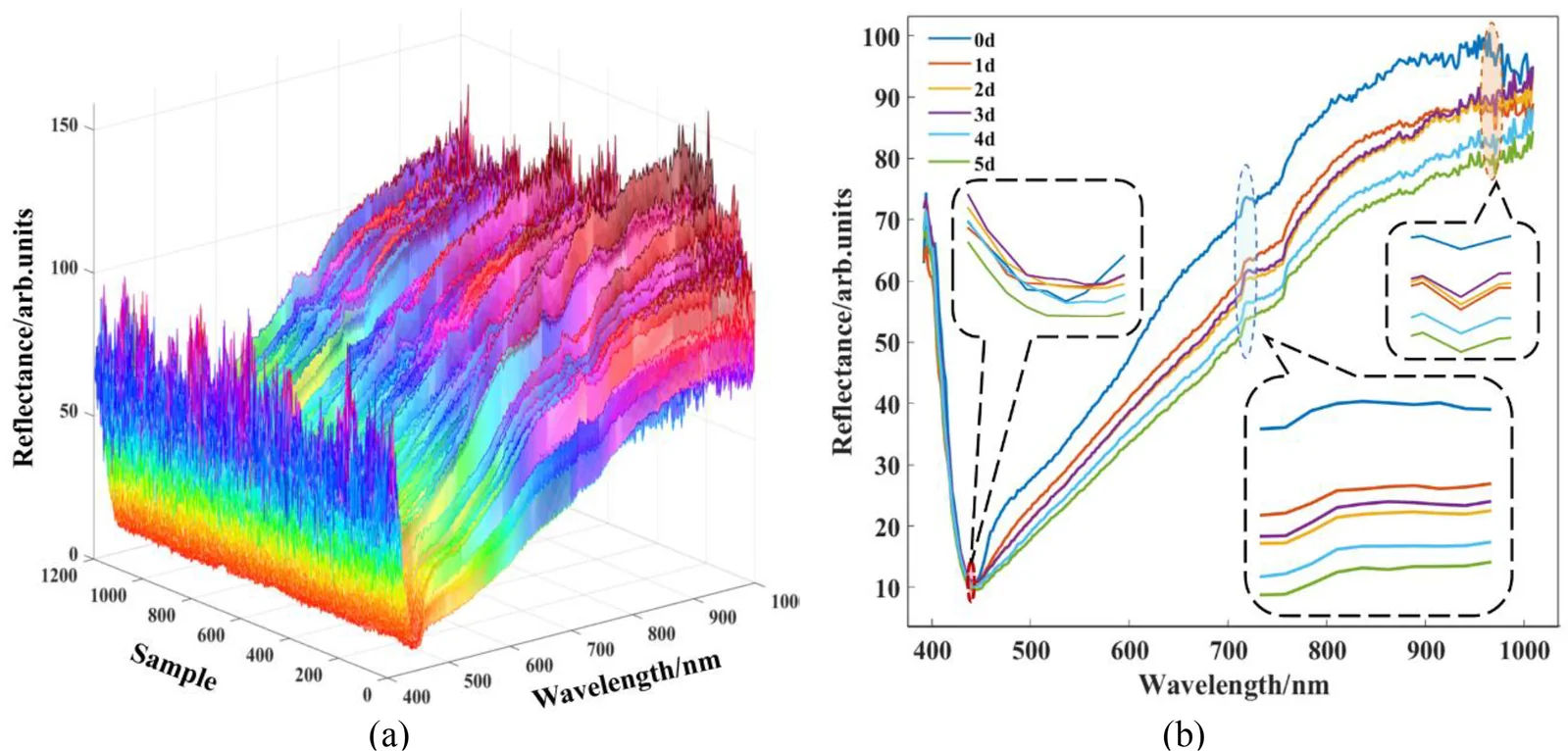
Original and mean spectra of rice seeds subjected to different aging durations. **(a)** Original spectra; **(b)** Mean spectra.

The primary hybrid indica rice dataset was used to characterize aging-related spectral variations and assess the effectiveness of the combined preprocessing strategy. **Figure 4** shows the original spectra and the average spectral curves of rice seeds subjected to different artificial aging durations. As shown in **Figure 4(a)**, the original spectra exhibited generally similar curve shapes across samples, indicating that rice seeds shared a consistent overall spectral response pattern. **Figure 4(b)** further illustrates the average spectral profiles of seeds aged for 0–5 d. In general, the 0 d control group exhibited the highest reflectance, whereas the 5 d aging group showed the lowest reflectance, especially in the 700–1000 nm region. Around 440 nm, all spectra displayed a distinct low-reflectance valley, which may be related to visible-light absorption by seed coat pigments and other compounds. With increasing aging time, slight changes in this region indicate that aging may alter the optical properties of the seed surface and associated biochemical constituents. Near 700–730 nm, the spectral curves showed slight spectral divergence among aging groups, suggesting that this region is sensitive to structural and compositional changes induced by seed deterioration. Around 970 nm, the reflectance decreased progressively with prolonged aging, which may be associated with changes in water status and storage substances such as starch and other carbohydrates. These wavelength-specific variations indicate that accelerated aging affects the internal biochemical composition and physical structure of rice seeds, resulting in distinguishable hyperspectral responses.

To verify whether the observed spectral differences were associated with aging-induced physiological deterioration, standard laboratory germination tests were conducted. As shown in **Table 1**, the germination percentage decreased continuously from 91.33% in the 0 d control group to 30.67% after 5 d of aging. This clear decline indicates that the accelerated-aging treatment generated a progressive deterioration gradient under controlled laboratory conditions. The correspondence between the decreasing germination percentage and the spectral changes shown in **Figure 4** suggests that hyperspectral information can be used to discriminate aging-induced vigor categories under the experimental conditions of this study.

**Table 1:** Germination percentages of rice seeds subjected to different accelerated-aging durations.

Aging duration (d)	0	1	2	3	4	5
Germination percentage (%)	91.33	80.67	72	58	45.33	30.67

**Figure 5:**
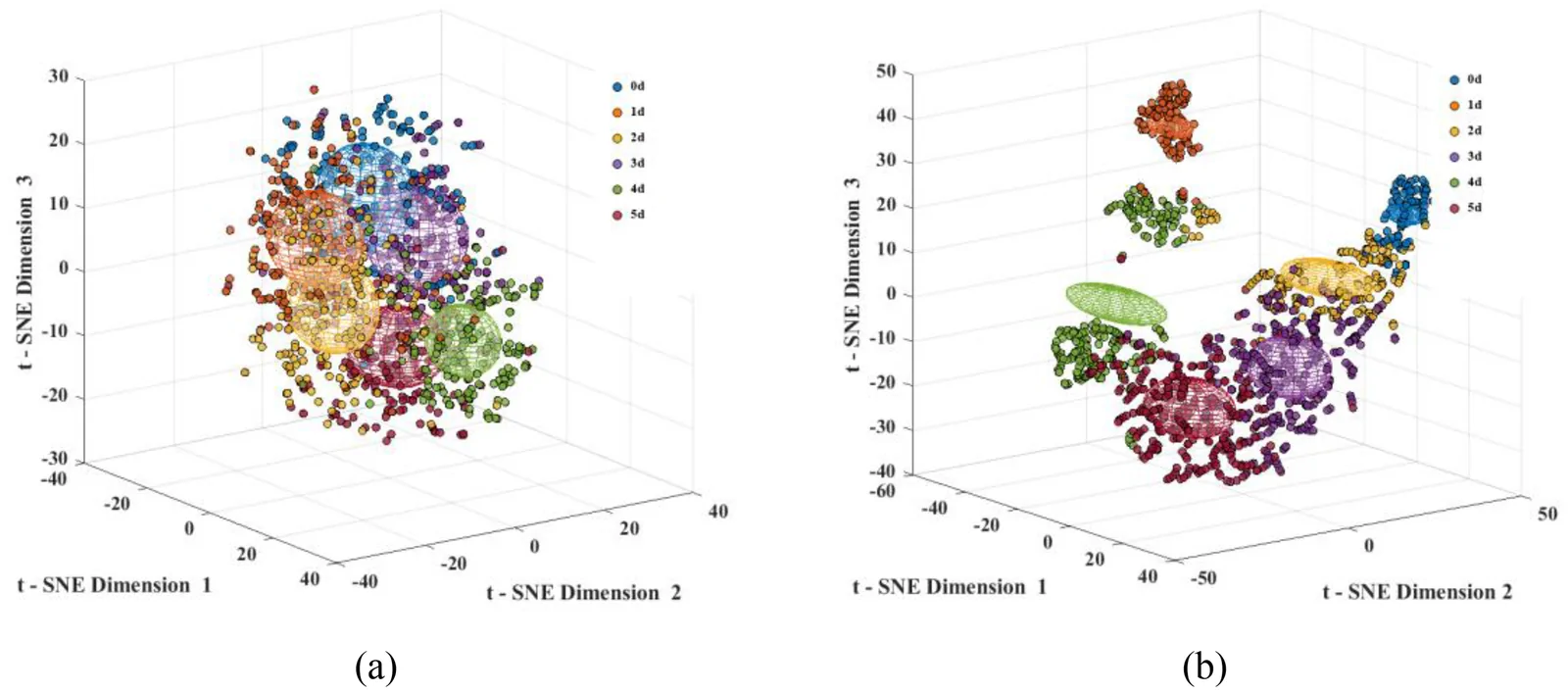
Three-dimensional t-SNE visualization of spectral features before and after preprocessing. **(a)** Original data; **(b)** AirPLS + SG + MSC-preprocessed data.

To reduce baseline drift, high-frequency noise, and scattering interference in the raw hyperspectral spectra, a combined preprocessing strategy consisting of AirPLS, SG smoothing, and MSC was applied. The feature distributions before and after preprocessing were visualized in three dimensions using t-distributed stochastic neighbor embedding (t-SNE), as shown in **Figure 5**. From **Figure 5(a)**, samples from different aging durations are highly intermixed, and the class boundaries are unclear. This indicates that baseline variation, random noise, and scattering effects in the raw spectra may obscure vigor-related spectral differences, making direct classification more difficult. After AirPLS + SG + MSC preprocessing as shown in **Figure 5(b)**, the distribution of samples becomes more structured. Spectra belonging to the same aging group show more compact clustering, while the separation among different aging groups is substantially improved. Although some neighboring vigor levels still exhibit partial proximity, the overall inter-class separability is clearly enhanced compared with the raw spectra. These results suggest that AirPLS helps correct baseline drift, SG smoothing suppresses high-frequency noise while retaining the main spectral profile, and MSC reduces scatter-related variations caused by seed surface morphology and optical path differences. Therefore, the combined preprocessing strategy improves the quality and consistency of the spectral data and provides more discriminative inputs for subsequent seed vigor classification.

### 3.2 Performance comparison of different models

To evaluate the performance of the proposed GCT-BCLN on the primary hybrid indica rice dataset, comparative experiments were conducted using representative machine learning and deep learning models, including SVM, Random Forest (RF), CNN, Transformer, and a CNN-Transformer fusion model. To ensure a fair comparison, the same dataset partitioning and preprocessing strategy were used for all models. For deep learning models, the Adam optimizer was adopted with an initial learning rate of 0.001, and a cosine annealing strategy was used to adjust the learning rate during training. The batch size was set to 32, and all models were trained for 100 epochs. For the GCT-BCLN model, the hyperparameters μ, ξ and τ in its composite loss function were set to 0.7, 0.01, and 0.005, respectively. These values were determined by grid search and cross-validation to balance the contributions of different loss terms.

The classification accuracies of different models on the training, validation, and test sets are summarized in **Table 2**. Overall, the deep learning models achieved better performance than the traditional machine learning methods. For example, CNN and Transformer obtained test accuracies of 0.9109 and 0.9384, respectively, outperforming RF and showing stronger ability to model nonlinear and high-dimensional hyperspectral data. However, the two single-architecture models showed different limitations. CNN is effective in extracting local spectral patterns, but its kernel-size-dependent receptive field restricts its ability to capture long-range inter-band dependencies. In contrast, Transformer can model global spectral relationships through self-attention, but it may be less sensitive to fine local spectral variations that are important for distinguishing adjacent vigor levels. The CNN-Transformer fusion model achieved a test accuracy of 0.9658, exceeding the standalone CNN and Transformer by 5.49% and 2.74%, respectively. This result indicates that integrating local convolutional features with global self-attention representations can improve seed vigor classification. Nevertheless, the conventional CNN-Transformer structure still relies mainly on serial and unidirectional feature fusion, which limits dynamic interaction between local and global representations. Compared with all baseline models, the proposed GCT-BCLN achieved the highest accuracy on the training, validation, and test sets, reaching 0.9951, 0.9523, and 0.9795, respectively. On the held-out test set, GCT-BCLN outperformed the CNN-Transformer model by 1.37% and exceeded SVM and RF by 6.28% and 13.70%, respectively. The improved performance can be attributed to the GRU-mediated closed-loop feedback mechanism. Specifically, CNN-extracted local spectral features are encoded by the GRU and supplied to the Transformer to enhance global spectral dependency modeling. Meanwhile, Transformer-derived global spectral information is fed back through the GRU to guide the CNN toward vigor-relevant spectral regions. This bidirectional interaction enables adaptive refinement of local and global representations, thereby improving the discrimination of subtle spectral differences among rice seed vigor levels.

**Table 2:** Classification accuracies of different models for rice seed vigor detection.

Models	Training	Validation	Test
SVM	0.9332	0.9042	0.9167
RF	0.8965	0.8571	0.8425
CNN	0.9591	0.9489	0.9109
Transformer	0.9542	0.9319	0.9384
CNN-Transformer	0.9854	0.9489	0.9658
GCT-BCLN	0.9951	0.9523	0.9795

**Figure 6:**
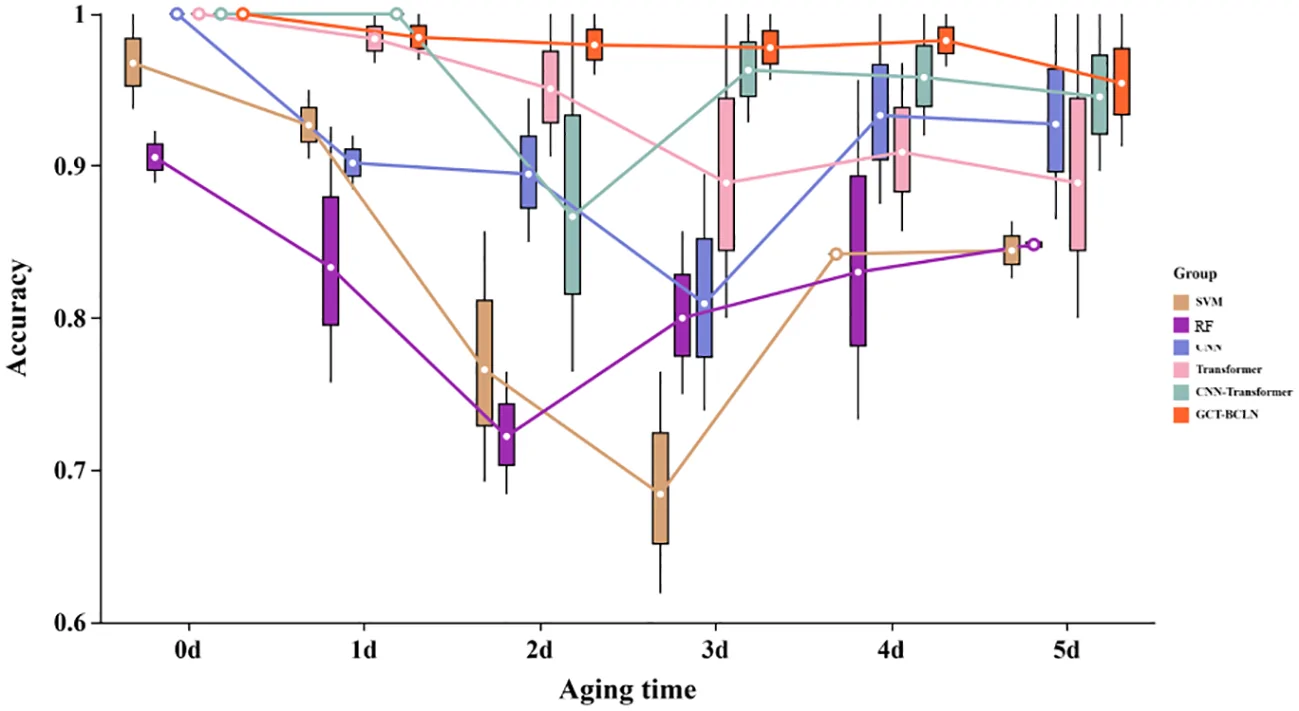
Box plots of classification accuracy for different models across rice seed aging durations.

To further evaluate the class-wise recognition ability of different models, precision, recall, and F1-score were calculated for each aging duration, as shown in **Table 3**. In addition, **Figure 6** presents the distribution of classification accuracy across aging stages. Overall, traditional machine learning models exhibited larger performance fluctuations. SVM and RF showed relatively low F1-scores at intermediate aging stages, especially for 2 d and 3 d samples, where the spectral differences between adjacent vigor levels were subtle and highly overlapping. This indicates that hand-crafted feature representations have limited ability to capture fine vigor-related spectral variations. Deep learning models generally achieved better performance, but their stability varied across aging stages. CNN obtained excellent performance for 0 d samples, with precision, recall, and F1-score all reaching 1.0000. However, its F1-score decreased to 0.8095 for 3 d samples, suggesting that local convolutional features alone are insufficient for transitional vigor states. Transformer improved the recognition of several classes through long-range spectral dependency modeling, but its F1-scores for 3 d and 5 d samples were both 0.8889, indicating that global attention alone may overlook local spectral details. The CNN-Transformer fusion model further enhanced overall performance by combining local and global features, achieving F1-scores above 0.94 for most aging stages. However, its F1-score for 2 d samples was only 0.8667, mainly due to the relatively low precision of 0.7647, suggesting that static serial fusion remains vulnerable when adjacent vigor levels are highly similar. In contrast, GCT-BCLN achieved the most balanced performance across all aging durations, with F1-scores of 1.0000, 0.9846, 0.9796, 0.9778, 0.9825, and 0.9545 from 0 d to 5 d, respectively. As shown in **Figure 6**, GCT-BCLN maintained high accuracy and better stability than the baseline models. Notably, for the challenging 2 d and 3 d samples, GCT-BCLN improved the F1-score by 11.29% and 1.48% over CNN-Transformer, respectively. These results suggest that the GRU-guided closed-loop feedback mechanism effectively enhances the interaction between CNN-based local spectral details and Transformer-based global spectral representations, thereby improving the discrimination of subtle vigor differences and maintaining robust performance throughout the seed aging process.

**Table 3:** Detection performance of different models on different seed vigor levels.

Models	Evaluation Metrics	Classification target					
		0d	1d	2d	3d	4d	5d
SVM	Precision	1.0000	0.9500	0.6923	0.7647	0.8421	0.8261
	Recall	0.9375	0.9048	0.8571	0.6190	0.8421	0.8636
	F1-score	0.9677	0.9268	0.7660	0.6842	0.8421	0.8444
RF	Precision	0.9259	0.8889	0.6842	0.8571	0.7333	0.8483
	Recall	0.9231	0.7576	0.7647	0.7500	0.9565	0.8514
	F1-score	0.9057	0.8333	0.7222	0.8000	0.8302	0.8449
CNN	Precision	1.0000	0.8846	0.8500	0.8947	1.0000	0.8649
	Recall	1.0000	0.9200	0.9444	0.7391	0.8750	1.0000
	F1-score	1.0000	0.9020	0.8947	0.8095	0.9333	0.9275
Transformer	Precision	1.0000	1.0000	1.0000	0.8000	0.9677	0.8000
	Recall	1.0000	0.9677	0.9062	1.0000	0.8571	1.0000
	F1-score	1.0000	0.9836	0.9508	0.8889	0.9091	0.8889
CNN-Transformer	Precision	1.0000	1.0000	0.7647	1.0000	1.0000	0.8966
	Recall	1.0000	1.0000	1.0000	0.9286	0.9200	1.0000
	F1-score	1.0000	1.0000	0.8667	0.9630	0.9583	0.9455
GCT-BCLN	Precision	1.0000	1.0000	0.9600	1.0000	1.0000	0.9130
	Recall	1.0000	0.9697	1.0000	0.9565	0.9655	1.0000
	F1-score	1.0000	0.9846	0.9796	0.9778	0.9825	0.9545

**Figure 7:**
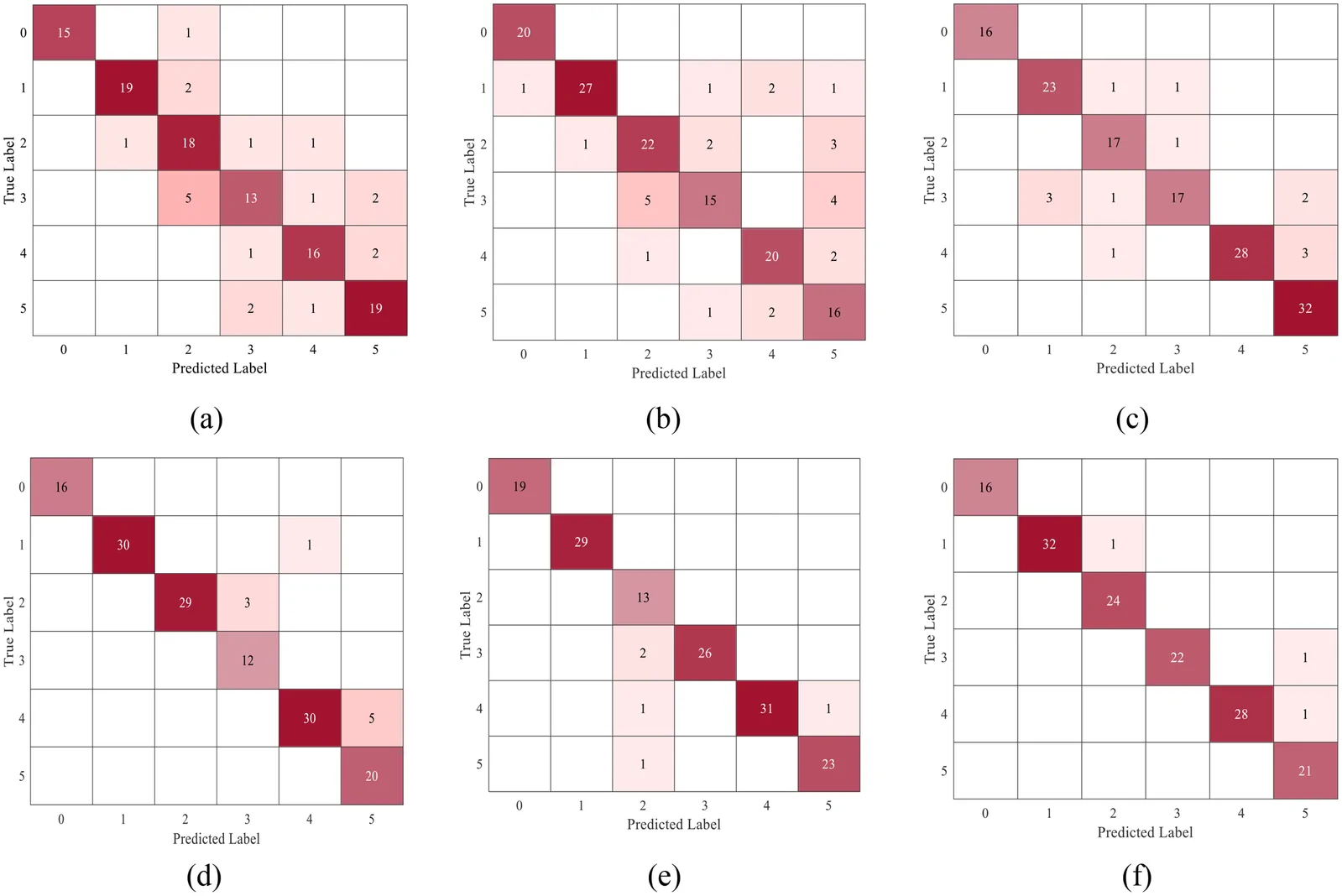
Confusion matrices of the classification models. **(a)** SVM; **(b)** RF; **(c)** CNN; **(d)** Transformer; **(e)** CNN-Transformer; **(f)** GCT-BCLN.

**Figure 8:**
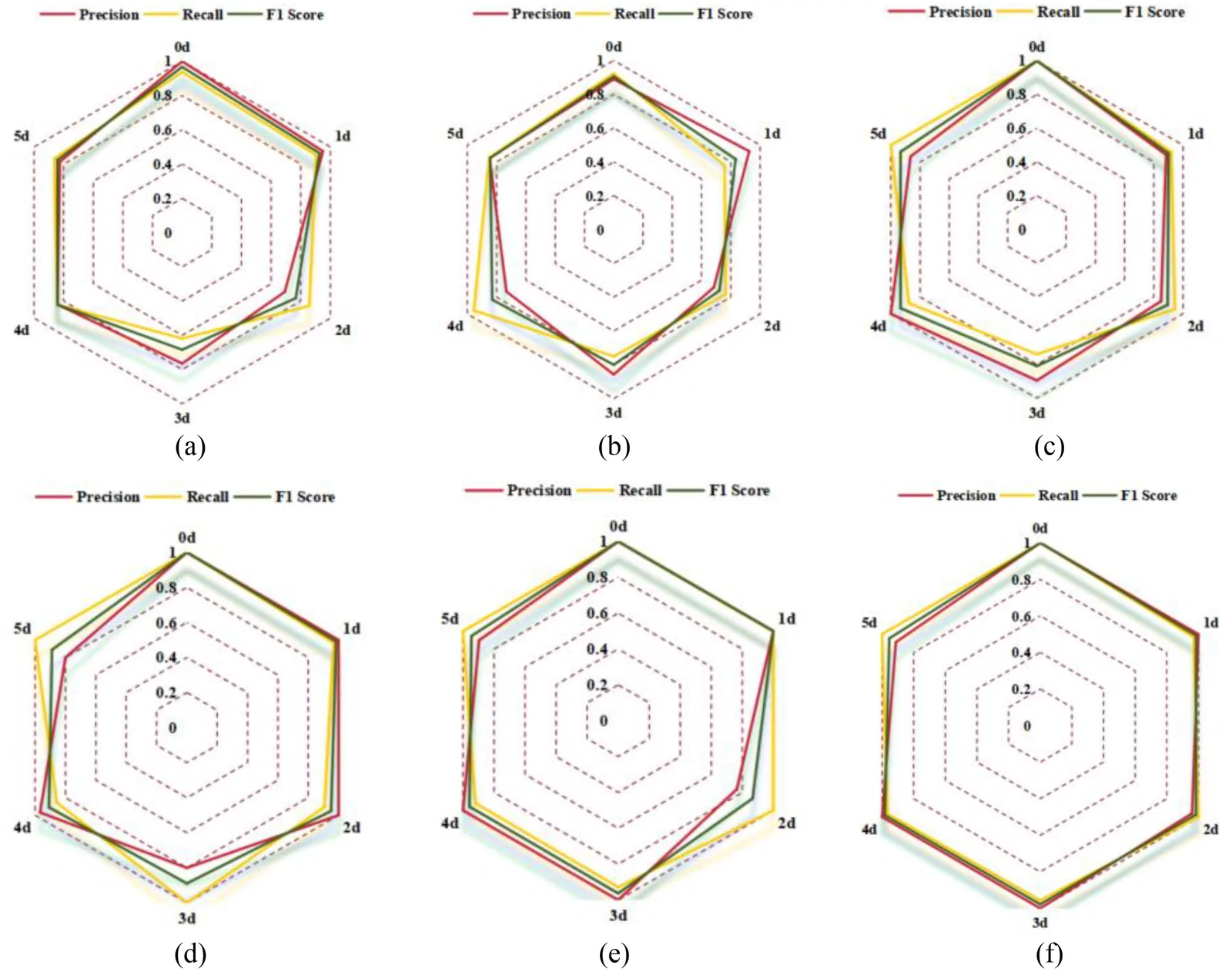
Radar charts of class-wise precision, recall, and F1-score for the classification models. **(a)** SVM; **(b)** RF; **(c)** CNN; **(d)** Transformer; **(e)** CNN-Transformer; **(f)** GCT-BCLN.

The confusion matrices in **Figure 7** and the radar charts in **Figure 8** further illustrate the classification consistency of different models across vigor levels. As shown in **Figure 7**, traditional models such as SVM and RF produced more errors, particularly among adjacent aging stages, indicating that they were less effective in distinguishing spectrally similar vigor levels. The CNN and Transformer models reduced part of these misclassifications, but errors still occurred in transitional stages such as 2 d and 3 d, where spectral differences were relatively subtle. The CNN-Transformer model further improved diagonal dominance, confirming the benefit of combining local and global features. However, some confusion remained between adjacent vigor classes. In comparison, GCT-BCLN showed the strongest diagonal dominance and the fewest off-diagonal errors among all models. Although a small number of samples were still misclassified between adjacent aging levels, the overall confusion was substantially reduced, especially for the challenging 2 d and 3 d samples. This indicates that the proposed GRU-guided closed-loop feedback mechanism improves the model’s ability to capture fine inter-class spectral variations. In **Figure 8**, the radar charts provide consistent evidence. Compared with SVM, RF, CNN, Transformer, and CNN-Transformer, GCT-BCLN maintained more balanced precision, recall, and F1-score across the six aging durations. Its radar profile was closer to the outer boundary and showed less fluctuation among different vigor levels, indicating better class-wise stability.

**Figure 9:**
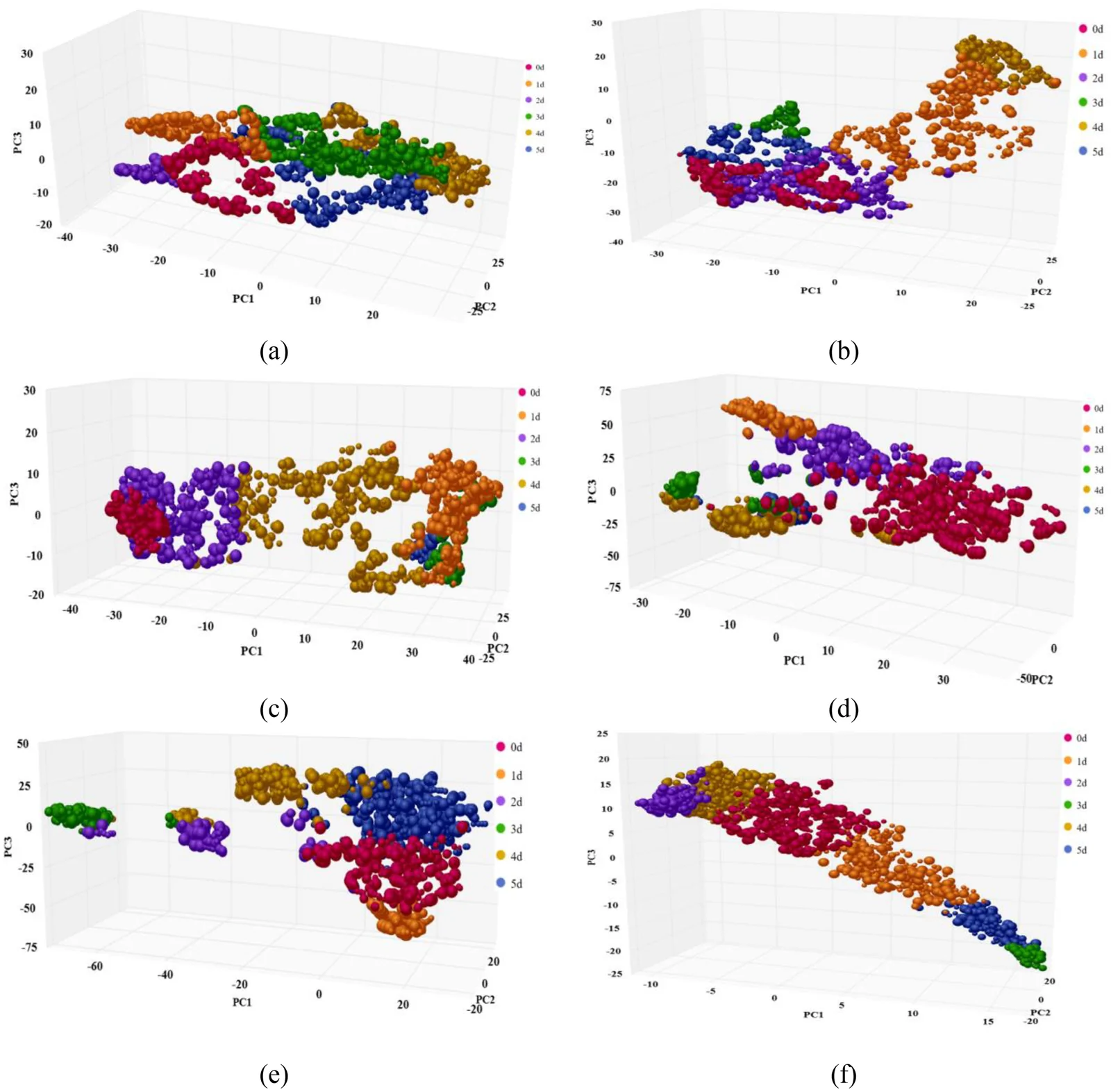
PCA visualization of feature representations extracted by the classification models. **(a)** SVM; **(b)** RF; **(c)** CNN; **(d)** Transformer; **(e)** CNN-Transformer; **(f)** GCT-BCLN.

To further evaluate the discriminative capacity of different models from the perspective of feature representation, principal component analysis (PCA) was applied to the features extracted from the final layer of each model, and the three-dimensional visualization results are shown in **Figure 9**. For traditional machine learning models, including SVM and RF, the feature distributions show substantial overlap among different aging levels, indicating limited ability to separate seeds with similar vigor characteristics. Although the CNN model presents a clearer clustering tendency than traditional methods, several aging groups still overlap, suggesting that local convolutional features alone are insufficient to fully distinguish subtle spectral differences among adjacent vigor levels. The Transformer model improves the overall separability of feature representations by modeling long-range spectral dependencies. However, some categories remain close to each other, implying that global attention alone may not adequately capture fine local spectral variations. The CNN-Transformer fusion model further improves feature organization, with more distinguishable clusters than the standalone CNN and Transformer models. Nevertheless, partial overlap and scattered samples are still observed among neighboring aging stages, indicating that static serial fusion has limited capacity for adaptive feature refinement. In contrast, the proposed GCT-BCLN produces a more structured feature distribution, with clearer inter-class separation and a more ordered arrangement among different vigor levels. In particular, adjacent aging stages show improved separation in the GCT-BCLN feature space. This indicates that the GRU-guided closed-loop feedback mechanism enhances the interaction between local spectral features and global spectral representations, allowing the model to learn more discriminative vigor-related spectral features. Overall, the PCA visualization further supports the superior classification performance of GCT-BCLN and demonstrates its stronger feature representation capability for rice seed vigor identification.

### 3.3 Evaluation of model performance across rice varieties

**Figure 10:**
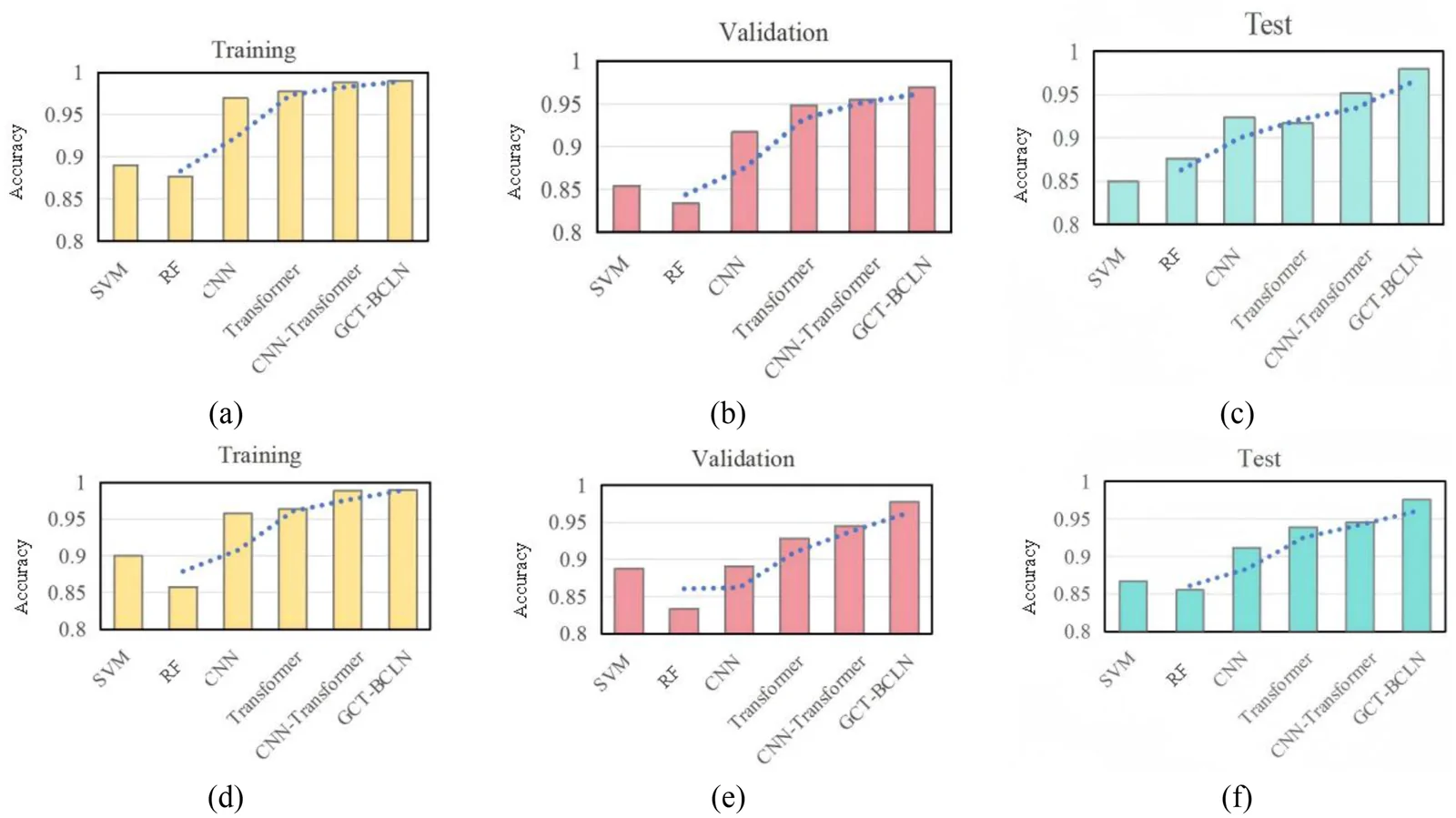
Performance comparison of the classification models across rice varieties. **(a–c)** Training, validation, and test accuracies for conventional japonica rice; **(d–f)** Training, validation, and test accuracies for glutinous japonica rice.

To further evaluate the performance of GCT-BCLN across different rice varieties under the same controlled protocol, additional experiments were conducted on two additional variety-specific datasets, namely conventional japonica rice and glutinous japonica rice. The same sample scale (1,200 seeds per variety), dataset partitioning, spectral preprocessing procedures, model architectures, and hyperparameter settings as those used for hybrid indica rice were adopted to ensure a consistent comparison. The classification accuracies of different models on the two rice varieties are summarized in **Table 4**, and the corresponding performance comparisons are illustrated in **Figure 10**. As shown in **Table 4**, GCT-BCLN achieved the best performance on both rice varieties. For conventional japonica rice, GCT-BCLN obtained training, validation, and test accuracies of 0.9901, 0.9688, and 0.9793, respectively, outperforming all baseline models. Its test accuracy was 2.76% higher than that of the CNN-Transformer model. For glutinous japonica rice, GCT-BCLN also achieved the highest accuracies, reaching 0.9899, 0.9772, and 0.9758 on the training, validation, and test sets, respectively. Compared with CNN-Transformer, the test accuracy increased by 3.03%. Traditional machine learning models, including SVM and RF, generally showed lower performance, with test accuracies mostly below 0.9. Standalone CNN and Transformer models performed better than traditional models, indicating their stronger ability to extract nonlinear spectral features. However, their performance was still lower than that of fusion-based models, suggesting that either local convolutional features or global attention features alone are insufficient for robust seed vigor classification across different rice varieties. The CNN-Transformer model achieved the second-best performance in most cases, confirming the benefit of combining local and global representations. Nevertheless, its static serial fusion strategy remained less effective than the closed-loop feature interaction used in GCT-BCLN. **Figure 10** provides a visual comparison of model accuracies on the training, validation, and test sets for conventional japonica rice (**Figures 10a–c**) and glutinous japonica rice (**Figures 10d–f**). Across both varieties and all data splits, GCT-BCLN consistently showed the highest bar values, while maintaining relatively small differences between validation and test performance. This indicates that the proposed model maintains stable classification performance on test samples within each variety. Overall, these results show consistent within-dataset performance across the examined varieties under the same controlled experimental protocol.

**Table 4:** Classification accuracies of different models for rice seed vigor detection across rice varieties.

Variety	Models	Training	Validation	Test
Conventional japonica rice	SVM	0.8901	0.8542	0.8500
	RF	0.8763	0.8339	0.8759
	CNN	0.9693	0.9169	0.9241
	Transformer	0.9772	0.9481	0.9172
	CNN-Transformer	0.9881	0.9550	0.9517
	GCT-BCLN	0.9901	0.9688	0.9793
Glutinous japonica rice	SVM	0.9000	0.8875	0.8667
	RF	0.8577	0.8332	0.8551
	CNN	0.9575	0.8907	0.9115
	Transformer	0.9639	0.9283	0.9387
	CNN-Transformer	0.9883	0.9453	0.9455
	GCT-BCLN	0.9899	0.9772	0.9758

## 4 Conclusion

To meet the demand for accurate and non-destructive rice seed vigor detection, this study combined hyperspectral imaging with deep learning and proposed a GRU-guided bidirectional closed-loop CNN-Transformer network (GCT-BCLN). A combined preprocessing strategy integrating AirPLS, SG smoothing, and MSC was first applied to reduce baseline drift, high-frequency noise, and scattering interference, thereby improving spectral data quality. On this basis, GCT-BCLN established a dynamic interaction between CNN-based local spectral feature extraction and Transformer-based global spectral modeling through a GRU-guided feedback mechanism, enabling more effective discrimination of seeds with different vigor levels. Experimental results showed that GCT-BCLN achieved a test accuracy of 0.9795 on the hybrid indica rice dataset, outperforming the CNN-Transformer model by 1.37% and exceeding traditional machine learning models by 6.28%–13.70%. In addition, the model also obtained test accuracies of 0.9793 and 0.9758 on conventional japonica rice and glutinous japonica rice datasets, respectively, indicating consistent performance across the three evaluated variety-specific datasets under the same controlled experimental protocol. These results suggest that the closed-loop feedback mechanism can effectively enhance the representation of subtle aging-related spectral differences and improve hyperspectral classification performance for rice seed vigor assessment. Overall, the proposed GCT-BCLN provides an effective technical approach for intelligent and non-destructive rice seed vigor assessment, with potential applications in seed quality evaluation, seed screening, and storage management. Nevertheless, this study still has certain limitations. The six classification targets were defined according to accelerated-aging duration rather than commercially defined vigor grades, and germination percentage was used as a group-level physiological indicator rather than an individual-seed vigor label. In addition, although three rice varieties were examined, each variety contained only 1,200 seeds, and all samples were obtained under controlled artificial-aging and laboratory hyperspectral-acquisition conditions. Moreover, deterioration induced by high-temperature and high-humidity accelerated aging may not fully reproduce the mechanisms occurring during natural storage or commercial seed handling. Under practical conditions, naturally deteriorated commercial seed lots may exhibit more complex physiological and spectral variability owing to differences in cultivar, production batch, harvest year, storage duration, storage temperature and humidity, transportation, and processing conditions.Therefore, further validation using larger independent datasets, naturally aged commercial seed lots from multiple production batches and storage histories, recognized laboratory seed-vigor assessments, and pilot-scale industrial conditions will be necessary before the reliability and practical applicability of GCT-BCLN can be established for industrial seed-vigor grading.

## Data Availability

The raw data supporting the conclusions of this article will be made available by the authors, without undue reservation.
